# Deep learning-based plane pose regression in obstetric ultrasound

**DOI:** 10.1007/s11548-022-02609-z

**Published:** 2022-04-30

**Authors:** Chiara Di Vece, Brian Dromey, Francisco Vasconcelos, Anna L. David, Donald Peebles, Danail Stoyanov

**Affiliations:** 1 Wellcome/EPSRC Centre for International and Surgical Sciences (WEISS), University College London, London, UK; 2 Department of Computer Science, University College London, London, UK; 3 Elizabeth Garrett Anderson Institute for Women’s Health, University College London, London, UK; 4 NIHR University College London Hospitals Biomedical Research Centre, University College London, London, UK

**Keywords:** Pose regression, Deep learning, Fetal ultrasound

## Abstract

**Purpose:**

In obstetric ultrasound (US) scanning, the learner’s ability to mentally build a three-dimensional (3D) map of the fetus from a two-dimensional (2D) US image represents a major challenge in skill acquisition. We aim to build a US plane localisation system for 3D visualisation, training, and guidance without integrating additional sensors.

**Methods:**

We propose a regression convolutional neural network (CNN) using image features to estimate the six-dimensional pose of arbitrarily oriented US planes relative to the fetal brain centre. The network was trained on synthetic images acquired from phantom 3D US volumes and fine-tuned on real scans. Training data was generated by slicing US volumes into imaging planes in Unity at random coordinates and more densely around the standard transventricular (TV) plane.

**Results:**

With phantom data, the median errors are 0.90 mm/1.17$^\circ$ and 0.44 mm/1.21$^\circ$ for random planes and planes close to the TV one, respectively. With real data, using a different fetus with the same gestational age (GA), these errors are 11.84 mm/25.17$^\circ$. The average inference time is 2.97 ms per plane.

**Conclusion:**

The proposed network reliably localises US planes within the fetal brain in phantom data and successfully generalises pose regression for an unseen fetal brain from a similar GA as in training. Future development will expand the prediction to volumes of the whole fetus and assess its potential for vision-based, freehand US-assisted navigation when acquiring standard fetal planes.

**Supplementary Information:**

The online version contains supplementary material available at 10.1007/s11548-022-02609-z.

## Introduction

**Fig. 1:**
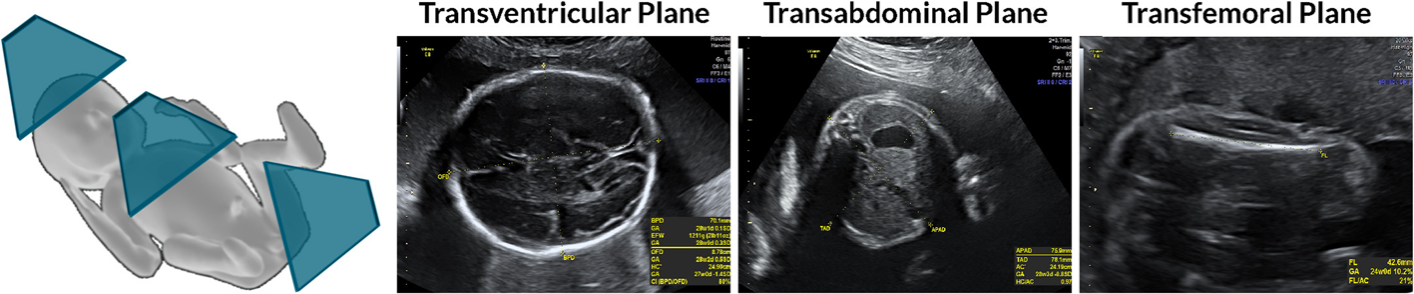
Three main standard US planes to evaluate the development of brain, abdominal and femoral structures. Their acquisition is subject to intra- and inter-operator variability

 In obstetrics, ultrasound (US) acquisition is a non-invasive, real-time and cost-effective diagnostic tool for monitoring mother and fetus throughout gestation [1]. Scientific committees promote international guidelines for obstetric US images [2] that must be acquired in particular standard planes (SPs) for diagnosis (Fig. 1). This allows for reliable measurements of specific structures and reduces inter- and intra-sonographer variability. The correct identification of SPs is essential in the second-trimester fetal anatomic survey to investigate the morphological characteristics of the fetus and detect abnormalities and deviations from the expected growth patterns. Sonographers may struggle to obtain good SPs for a variety of reasons, including inexperience, limited training, time limitations and fetal movement [3, 4]. The primary training challenge faced by all novice sonographers is not related to knowledge of anatomy or familiarity with the US machine interface. Rather, the manual navigation of the probe towards acquiring SP requires the sonographer to build a three-dimensional (3D) map of the fetus from dynamic two-dimensional (2D) sectional views while handling the probe. The majority of trainees learn on actual patients under the direct supervision of an expert. Although US simulators have been developed in recent years, trainee engagement has been limited due to competing time priorities [5].

This challenge in clinical training could be addressed with a US navigation system that guides the sonographer towards obtaining SPs with reference to fetal anatomy. In this paper, we propose a deep learning (DL)-based plane localisation system to estimate the six-dimensional (6D) pose of arbitrarily oriented US planes with respect to the centre of the brain volume. Our method is purely image-based and, therefore, does not require tracking sensors. Additionally, it is also not a classic slice-to-volume registration method, *i.e.* it does not require a previously acquired 3D volume of the same subject being scanned. Instead, we predict the pose relative to a generalised brain centre, *i.e.* a stable anatomical brain point across the different, pre-aligned volumes, where training and test data belong to different subjects. Our contributions are as follow:

- To the best of our knowledge, it is the first work estimating any 6D pose (3D translation and rotation: $t_x, t_y, t_z, \alpha _x, \alpha _y, \alpha _z$) of a US plane relative to the fetal brain centre solely based on 2D scans.
- We formulate 6D pose estimation as deep neural network regression, representing rotations with a continuous 6D representation [6] since conventional rotation representations (Euler angles, quaternions, axis-angle) are not reliable in this setting.
- We developed a 3D environment using the Unity engine for automated generation of supervised data to train our network using pre-acquired 3D US volumes of both phantom and real fetuses.
- We provide a quantitative analysis demonstrating that our method works reliably on phantom data and generalises better to unseen real fetus scans if the gestational age (GA) of the considered fetus is not too far from the one used for training (23 weeks).
- We release our trained models and the 3D US phantom volumes with transventricular (TV) SP slice and pose annotations.

**Fig. 2:**
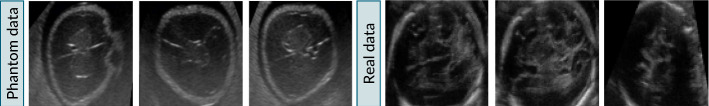
Examples of automatically generated supervised data to train our network using pre-acquired 3D US volumes of both phantom and real fetuses.

## Related work

The pose of a slicing plane with respect to a volume can be estimated with traditional approaches such as *feature-based* and *intensity-based* slice-to-volume registration or convolutional neural network (CNN)-based methods. In traditional approaches, iterative numerical optimisation maximises intensity-based similarity metrics or minimises the distance between registered point features [7, 8]. However, the cost functions associated with these metrics are frequently non-convex and require a reliable initialisation. They are also computationally costly and, more importantly, require having a 3D volume of the subject being scanned beforehand, which is not suitable for a point-of-care fetal US application. With the increased interest in DL, new approaches have been proposed to address the ill-posed slice-to-volume registration problem using CNNs [9, 10]. 3D pose estimation methods based on CNN are classifiable into two groups. The first includes models that predict keypoints used to find the orientation [11, 12]. The second group comprises models predicting the object pose directly from images [13, 14]. Works like [11, 15] demonstrated that DL metrics slightly outperform patch features and local image intensity, which are typically employed in slice-to-volume registration. Pose estimation has been primarily approached as a classification problem, with the pose space being discretised into bins [13, 14]. Conversely, Mahendran et al. [16] have modelled the 3D object pose estimation as a regression problem, proposing a deep CNN to estimate rotation matrices with a new geodesic distance-based loss function. In fetal magnetic resonance imaging (MRI) [17] and fetal US [18], learning-based approaches have also been proposed. Namburete et al. [18] formulated the alignment of fetal US as a one-coordinate position estimation and a 3-class slice plane classification. They trained their CNN using the negative likelihood loss to simultaneously predict slice location and brain segmentation. Hierarchical learning has been proposed for pose estimation in works such as [11, 19]. Here, the six dimensions of the parameter space were partitioned into three areas to separately learn the regression function based on in-plane and out-of-plane rotations as well as on out-of-plane translations hierarchically in order to speed up slice-to-volume rigid registration and improve its capture range. However, the pose estimation was based on a 2D-projected image representation of objects, leading to limited rotations. Li et al. [20] proposed a new approach for standard plane detection in 3D fetal US using a CNN to regress a rigid transformation iteratively comparing different transformation representations. In [21], Salehi et al. used a CNN to estimate the 3D pose (rotation and translation) of arbitrarily oriented MRI slices based on their sectional image representations for registration purposes. To this aim, they devised a regression problem based on the angle-axis representation of 3D rotations.

Deep learning regression of 6D pose, and in particular 3D rotations, is a widely studied topic beyond the medical field. Different rotation representations have been used in this context. Works like [22] adopted quaternions for regression, which are free from singularities but have an antipodal problem. This issue is also shown in [23], where the authors reported a high percentage of errors between 90$^\circ$ and 180$^\circ$. Axis-angle representation has also been used [24] to estimate the 6D pose of object instances starting from RGB images, depth maps or scanned point clouds. However, Zhou et al. [6] showed that any rotation representation in 3D with less than five dimensions is discontinuous in the real Euclidean space, making them harder to learn. Empirically, the network converges but produces large errors for specific rotation angles. To cope with this limitation, they proposed a new continuous representation for the *n* dimensional rotations *SO*(*n*), the “6D-loss”, obtained through projection and normalisation of the first two rows of each rotation matrix and continuous for all elements in *SO*(3): $\mathcal {L}_{6D}~=~\left\| (\tilde{R}_{:,1:2}/\left\| \tilde{R}_{:,1:2} \right\| _2) - (R_{:,1:2}/\left\| R_{:,1:2} \right\| _2) \right\| _2$. Empirical results suggest that continuous representations (5D, 6D and vector-based) outperform discontinuous ones (Euler angles, quaternions, axis-angle) and are more suited for the regression task.

## Methods

### Dataset generation

**Phantom fetal data** We acquired six brain volumes on a 23-week GA fetus US examination phantom by MediScientific Ltd., Roecliffe, York, UK^1^ (whole phantom: 40$\times$29$\times$22 $\mathrm{cm}$; fetus: 26 $\mathrm{cm}$). The volumes $p_{j}$, with $j=1,...,6$ indicating the acquisition number, were acquired using the Voluson^TM^ E10 BT18 Women’s Health Ultrasound System^2^ and the eM6C 4D 3D US probe,^3^ both by General Electric (GE) Healthcare, Chicago, IL, USA. All volumes were processed to be isotropic with voxel size of 0.5$\times$0.5$\times$0.5 $\mathrm{mm}$ and average size of 249$\times$199$\times$160 $\mathrm{mm}$ (*coronal*$\times$*axial*$\times$*sagittal*, actual size of the acquired volumes). They were registered using the general registration (BRAIN) module available in 3D Slicer^4^ with a similarity registration phase (7 degrees of freedom in total). To generate training data for our models, we extract image slices with a purpose-built program that contains the acquired US volumes within the game engine Unity.^5^ The slices were generated by applying rotation and translation to a plane with a starting in the centre of the volume generated with a uniform random distribution within a fixed range to avoid slices with poor overlap with the volume. The synthetic images obtained by slicing the volume were saved along with their pose with respect to the volume centre (fetal brain). This provides an automated way of generating a high amount of training data with reliable ground truth labels. An experienced sonographer annotated the position of the TV SP by directly manipulating a slicing plane within Unity and chose the translation and angle sampling intervals to avoid sampling of planes at the edges of the volume containing no information. The nearby planes were generated by applying small random rotations and translations (uniform distribution). Specifically, the acquisition interval between two planes was decreased from 0.1 to 0.001 for translation (Unity environment, with coordinates normalised between −1 and 1 so that the pose regression works in a fixed, normalised range, independent of the real brain size in $\mathrm{mm}$) and from 7.9$^\circ$ to 1.9$^\circ$ for rotation. We acquired 18047 planes with random orientation per volume and 725 around the TV SP.

**Real fetal data** We analysed the generalisation capability of our method on a dataset of seven real fetal brain US volumes with a GA ranging from 21 to 25 and 39 weeks [25] (singleton pregnancy with no abnormal findings)^6^ obtained from different fetuses ($r_{i}$, with $i=1,...,7$). The average size of the volumes is 249$\times$174$\times$155 $\mathrm{mm}$; the voxel size is 0.5$\times$0.5$\times$0.5 $\mathrm{mm}$. We acquired 22029 images for each volume (20699 at random coordinates and 1330 around the TV SP) following the same procedure used for phantom data.

Figure 2 shows an example of supervised phantom and real data automatically generated with our 3D Unity-based environment.

**Fig. 3:**
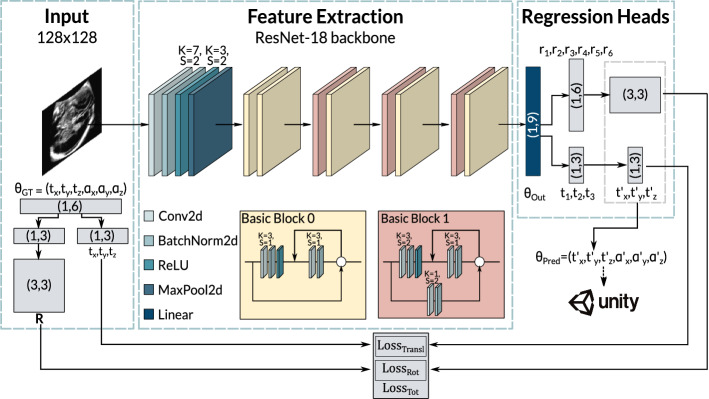
Diagram of the proposed pose regression network. During training, it receives US images sliced from the volume and their 6D pose $\theta _{\mathrm{GT}} = (t_x, t_y, t_z, \alpha _x, \alpha _y, \alpha _z)$ relative to the centre of the fetal brain. It outputs a pose prediction as $\theta _{\mathrm{Out}}=(t_1, t_2, t_3, r_1,..., r_6)$, from which a rotation matrix $\mathbf{R}'$ and a translation vector $t_{\mathrm{Pred}} = (t'_x,t'_y,t'_z)$ are extracted for the loss function. This pose is also represented as $\theta _{\mathrm{Pred}} = (t'_x, t'_y, t'_z, \alpha '_x, \alpha '_y, \alpha '_z)$ for visualisation in Unity. K and S refer to the kernel size and the stride

### Network architecture

As proposed in [21], we used an 18-layer residual CNN (ResNet-18) [26] as a backbone for feature extraction with the pre-trained ImageNet weights [27].We modified the network by re-initialising the fully connected layer based on the representation’s dimension and adding a regression head to output the rotation and translation representations directly. An overview of the proposed framework is presented in Fig. 3. The network receives the US image *I* (128$\times$128) obtained by slicing the volume and its 6D pose with respect to the centre of the fetal brain US volume $\theta _{GT}~=~(t_x, t_y, t_z, \alpha _x, \alpha _y, \alpha _z)$. We use this information as the ground truth label for network training and validation. The CNN learns to predict the 6D pose with respect to the same point $\theta _{\mathrm{Pred}}=(t'_x, t'_y, t'_z, \alpha '_x, \alpha '_y, \alpha '_z)$. Specifically, the network first outputs a vector of nine parameters $\theta _{Out} = (t_{1}, t_{2}, t_{3}, r_{1}, ..., r_{6})$; the first three are used for the translation and the last six for the rotation. Then, $r_{1}, ..., r_{6}$ are used internally by our CNN to reconstruct the rotation matrix $\mathbf{R} '$ in the forward pass. To do so, we employ Gram-Schmidt process and construct orthonormal basis from two vectors. If the neural network outputs two vectors $\vec {v_1}$ and $\vec {v_2}$, then 3D rotation matrix (R’) can be obtained as follows: $\vec {e_1} = \frac{\vec {v_1}}{||\vec {v_1}||};~\vec {e_2}~=~\frac{\vec {u_2}}{||\vec {u_2}||}, \vec {u_2}~=~\vec {v_2}-(\vec {e_1}\cdot \vec {v_2})\vec {e_1} \longrightarrow R' = (\vec {e_1}~\vec {e_2}~\vec {e_1}\times \vec {e_2})$.

### Loss function details

For both translation and rotation we used as loss the mean squared error (MSE) between predicted ($\mathbf{t} '$,$\mathbf{R} '$) and ground truth ($\mathbf{t}$,$\mathbf{R}$) values

1 $$\begin{aligned} \mathcal {L}_{\mathrm{Translation}} = \frac{1}{N}\sum _{t=1}^{N}\left\| \mathbf{t} '-\mathbf{t} \right\| _2,~\mathcal {L}_{\mathrm{Rotation}} = \frac{1}{N}\sum _{t=1}^{N}\left\| \mathbf{R} '-\mathbf{R} \right\| _2 \end{aligned}$$

where *N* denotes the total number of images *I* within one training epoch, $\mathbf{t} '$ denotes the predicted translation component and $\mathbf{t}$ the label. $\mathbf{R}$ is the 3$\times$3 rotation matrix obtained from the ground truth rotation vector $\mathbf{r} ~=~(r_x,r_y,r_z)$ and $\mathbf{R} '$ is the 3$\times$3 rotation matrix obtained from the six parameters $r_1,...,r_6$ as the output of the networks. The total loss function is then computed as: $\mathcal {L}_{\mathrm{Total}} = \mathcal {L}_{\mathrm{Rotation}} + \lambda \mathcal {L}_{\mathrm{Translation}}$ where $\lambda$ is a hyperparameter to balance between the rotation loss $\mathcal {L}_{\mathrm{Rotation}}$ and the translation loss $\mathcal {L}_{\mathrm{Translation}}$.

## Experiments and results

Our framework is implemented in *PyTorch* and trained using a single Tesla® V100-DGXS-32GB GPU of an NVIDIA® DGX station. The network was trained for 50 epochs with a batch size of *K* = 100 using Adam optimiser, with a learning rate of 0.0001 and exponential decay rates $\beta _1$ and $\beta _2$ of 0.9 and 0.999, respectively. We tested three different values for the hyperparameter $\lambda$ that weights rotation and translation ($\lambda$ = 0.1, 0.01, 0.001). Since $\lambda =$ 0.01 provides the best balance between translation and rotation errors (Table 1 in Supplementary Material), we used this value for the experiments on both phantom and real data. We choose the best model weights considering MSE obtained on the validation set (20% of the training set).

### Comparison experiments

Regarding the rotation representation, we also tried implementing regression with Euler angles and quaternions, but they produced large errors for specific rotation angles on training data. Therefore, we only concentrate on the 6D continuous representation for rotation.

Our study is divided into two different experiments. As before, we indicate the volumes considered in the experiments as $p_{j}$ (phantom data) and $r_{i}$ (real data), where *j* indicates the acquisition number and *i* the fetus. Images were resized to 128$\times$128, preserving the same aspect ratio, and cropped and centred to avoid visible sharp edges that could cause overfitting.

**Table 1:** Translation and rotation errors of our method for test planes acquired at random coordinates (Test RP), and around TV SP (Test SP). Norm: Euclidean distance, GE: Geodesic Error. $p_j$ and $r_i$ refer to phantom and real volumes, respectively, where *i* indicates the fetus considered. For tests on real data, $w_i$ indicates the GA

Initial weights	Training data	Testing data	Interval	Translation Norm [mm]			Rotation GE [deg]		
				Median	Min	Max	Median	Min	Max
ImageNet	Phantom ($p_1, p_2, p_3, p_4$)	Phantom ($p_5, p_6$)	Test RP	0.90	0.01	53.47	1.17	0.04	20.85
			Test SP	0.44	0.02	10.43	1.21	0.13	137.78
Phantom	Real ($r_1 - w_{23}$)	Real ($r_2 - w_{21}$)	Test RP	9.94	0.26	37.58	30.58	0.54	155.3
		Real ($r_3 - w_{22}$)	Test RP	10.74	0.29	43.03	30.81	0.91	146.1
		Real ($r_4 - w_{23}$)	Test RP	10.39	0.32	39.08	21.94	0.43	137.22
		Real ($r_5 - w_{24}$)	Test RP	17.76	0.24	58.44	37.93	1.39	131.3
		Real ($r_6 - w_{25}$)	Test RP	18.80	0.25	50.59	42.23	1.82	108.2
		Real ($r_7 - w_{39}$)	Test RP	17.15	1.31	61.39	34.43	0.71	159.64

**Experiment 1** We investigated two different scenarios.

1. Training ($p_1, p_2, p_3, p_4$, 75088 images) and testing ($p_5, p_6$, 37544 images) on phantom data; initialisation with weights from ImageNet;
2. Training and testing on real data; initialisation with weights from the phantom. The model is trained on one fetus ($r_{1}$, 22029 images) with a GA of 23 weeks and tested on six real volumes obtained with a single acquisition of different fetuses ($r_2,..., r_7$) ranging from a GA of 21 to 39 weeks to understand how well the model generalises over different shapes and sizes.

The obtained models were used to perform the inference on the test sets dividing them into two subgroups: (a) random planes (*Test RP*) and (b) planes around TV SP (Test SP). To evaluate the translation results we employed the Euclidean distance between the two planes, reported in $\mathrm{mm}$. For rotation, we display errors as the geodesic distance to ground truth in $\mathrm{degrees}$, more suitable for the geometric interpretation of the distance between two 3D rotations and defined as $\mathrm{Error}_{\mathrm{Rotation}} = \arccos ((\mathbf{R} ''_{00}+\mathbf{R} ''_{11}+\mathbf{R} ''_{22}-1)/2)$, where $\mathbf{R} ''~=~\mathbf{R} '^{-1}$. The median, maximum and minimum errors are reported in Table 1. The average inference time is 2.97 ms per plane.

**Fig. 4:**
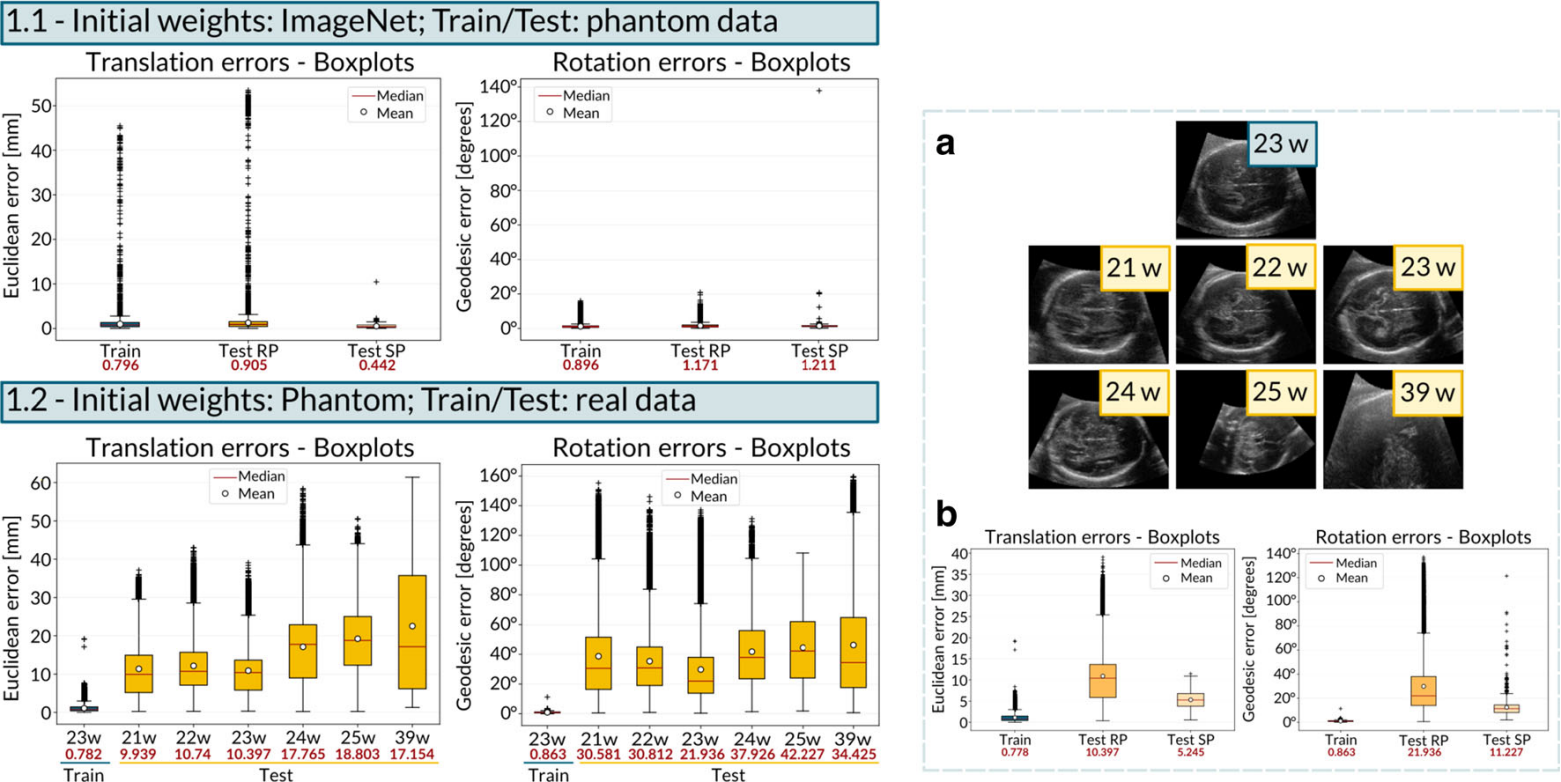
Left: Translation and rotation error distributions in phantom (1.1) and real US data (1.2). Test RP refers to test planes acquired at random coordinates, whereas Test SP refers to test planes acquired around the annotated TV SP. Right: **a** shows the central slice of fetal brain volumes used for training (blue labels) and testing (yellow labels) in Experiment 1.2. **b** RP and SP comparison for a GA of 23 weeks, *i.e.* the best aligned volume

Figure 4 reports the translation and rotation error distributions.

**Fig. 5:**
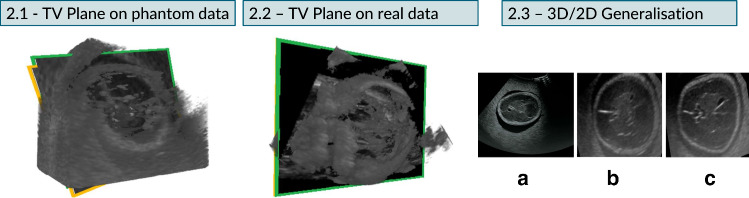
TV SP prediction performed on phantom (2.1) and real (2.2) US data. The green and orange boxes indicate the ground truth and the prediction, respectively. The ground truth pose of the TV SP was manually annotated by an experienced sonographer within the Unity environment. In 2.3, **a** is the TV SP acquired on the phantom with a 2D probe, **b** is where the predicted plane intersects the phantom 3D US volume (scanned with 3D US probe), and **c** is the SP annotation in the 3D US volume, which is similar to the prediction

**Experiment 2** In the second set of experiments, we performed a sanity test using the manually annotated TV SPs. The sectional images were saved and fed into the network to estimate their pose. We plotted back the two planes within the volume into Unity to visually evaluate the distance between the annotated TV SPs and the predicted ones for both phantom (2.1) and real data (2.2). To understand if the model can generalise to 2D acquisitions, we fed into the network a SP acquired on the phantom with a 2D probe (4C-RS Ultrasound Probe/Transducer,^7^ by GE Healthcare, Chicago, IL, USA) (2.3). The sonographer compared the appearances of the predicted plane and the externally annotated TV SP, slicing the 3D volume in Unity at the predicted and annotated coordinates, respectively. He confirmed that they contained the same anatomical information. The obtained results are shown in Fig. 5.

## Discussion and conclusions

This paper introduces a regression CNN to predict the 6D pose of arbitrarily oriented planes slicing the fetal brain US volume without the need for real ground truth data in real-time or 3D volume scans of the patient beforehand. Estimating the pose solely relative to the anatomy ensures independence from the considered reference frame. However, to achieve this, we need to make implicit assumptions about fetal anatomy, namely that the location of brain structures relative to a normalised brain volume is stable across different fetuses. As observed in the experiments, the GA has an impact on this assumption. An effective solution may need to be designed around a specific age range. Fortunately, fetal examinations are standardised in time (1st, 2nd, 3rd trimester), enabling fine-tuning to these specific intervals. A second assumption is that the scanned fetus is healthy, and therefore brain anomalies would present a challenge. On the other hand, our proposed model would find its best performance in clinical training with phantom simulation. In this case, our method provides accurate pose estimations, as demonstrated by our phantom experiments, and may enable assisted 3D navigation and skills assessment when using physical phantoms. Although errors are still high on real data, better results could be achieved either by extending the training dataset or using our method to get an initial rough alignment and combine it with approaches for position refinement. Future work could extend the same concept to other anatomical regions such as the abdomen, expand the network input to video clips, and use the temporal context for increased robustness.

## Supplementary Information

Below is the link to the electronic supplementary material.

Supplementary file 1 (pdf 5486 KB)

## Footnotes

1 US-7a Fetus Ultrasound Examination Phantom (MediScientific Ltd., Roecliffe, York, UK)

2 VolusonTM E10 BT18 Women’s Health Ultrasound System (GE Healthcare, Chicago, IL, USA).

3 eM6C 4D 3D US probe (GE Healthcare, Chicago, IL, USA).

4 3D Slicer

5 Unity Real-Time Development Platform

6 Refer to www.datavers.nl for details.

7 4C-RS Ultrasound Probe/Transducer (GE Healthcare, Chicago, IL, USA)
