# Supplementary material for: Deep learning-based plane pose regression in obstetric ultrasound
Source: Int J Comput Assist Radiol Surg. 2022 Apr 30;17(5):833–9. doi: 10.1007/s11548-022-02609-z (PMC9110476; doi:10.1007/s11548-022-02609-z)
Supplement: Supplementary file 1 — (pdf 5486 KB) [file 11548_2022_2609_MOESM1_ESM.pdf]

Deep Learning-based Plane Pose Regression in Obstetric Ultrasound

Supplementary Material

Chiara Di Vece[1,2*], Brian Dromey[1,3], Francisco Vasconcelos[1,2], Anna L. David[3,4], Donald Peebles[3,4] and Danail Stoyanov[1,2]

[1*]Wellcome/EPSRC Centre for International and Surgical Sciences (WEISS), University College London, London, UK.
[2]Department of Computer Science, University College London, London, UK.
[3]Elizabeth Garrett Anderson Institute for Women's Health, University College London, London, UK, London, UK.
[4]NIHR University College London Hospitals Biomedical Research Centre, University College London, London, UK.

*Corresponding author(s). E-mail(s): chiara.divece.20@ucl.ac.uk; Contributing authors: b.dromey@ucl.ac.uk; f.vasconcelos@ucl.ac.uk; a.david@ucl.ac.uk; d.peebles@ucl.ac.uk; danail.stoyanov@ucl.ac.uk;

Link

All the files mentioned in this document can be found at the following link: https://github.com/surgical-vision/ipcai2022-pose-regression.

Dataset Generation

Starting from a slice located in the centre of the volume and aligned with x,y axes (*i.e.*, all coordinates are zero), we generate random planes by applying a random rotation and translation to this zero plane. The random rotations and translations are generated with a uniform random distribution within a

Table 1 Intervals chosen by the experienced sonographer for the random sampling of the standard planes to avoid planes with no informative content. The translation is then normalised between -1 and 1. The normalization was applied because it enables us to have a pose regression in a fixed, normalized range, independent of the real brain size in *mm*

	**Rotation** [degree]	**Translation** [Unity unit]
**x**	-15/+15	-0.1/+ 0.1
**y**	-15/+15	-0.01/+0.01
**z**	0/180	-0.15/+0.15

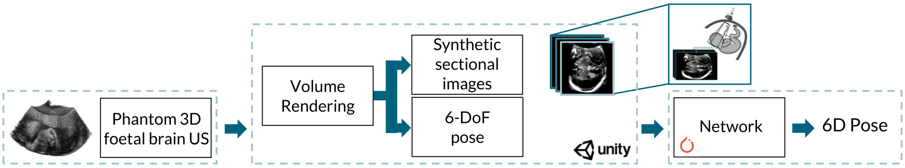

Fig. 1 Pipeline to train and test the network. The 3D fetal brain ultrasound volume was reconstructed into Unity, and synthetic sectional (slice) image representations were generated and saved along with their 6D pose (translation and rotation) relative to the centre of the fetal brain ultrasound volume. These images are fed into the network to output the estimated slice 6D pose (translation and rotation) relative to the same point

fixed range to avoid slices with poor overlap with the volume. We have added a video showing the sampling procedure in the folder `videos`, as an image would have been too dense. On the other hand, to obtain the standard planes, the exact coordinates of the transventricular standard plane were annotated by an experienced sonographer by directly manipulating a slicing plane within Unity. We then generate nearby planes once these coordinates are known by applying small random rotations and translations to its original coordinates, again generated following uniform distribution in each of the three coordinates. Table 1 reports the ranges of acquisition for both random and standard planes.

Additional Experiments

This section reports the results for all the additional experiments made in this study. The pipeline to train and test the network is shown in Figure 1. We remind the reader that we refer to the used volumes as follows: $p_{i,j}$ and $r_{i,j}$ refer to fetal brain volumes acquired from an examination phantom and real fetuses, respectively. Specifically, i indicates the fetus number and j the acquisition number. For phantom volumes $j = 1, ..., 6$, whereas for real volumes $i = 1, 2$.

Tuning of the λ hyperparameter

As explained in the paper, we tested three different values for the hyperparameter λ that weights rotation and translation ($\lambda = 0.1, 0.01, 0.001$). Since $\lambda = 0.01$ provides the best balance between translation and rotation errors (Table 2), we used this value for the experiments on both phantom and real data. We

Table 2 Test of different values for the hyperparameter λ weighting translation and rotation error on phantom data. Norm: Euclidean Distance, GE: Geodesic Error

	**Translation - Norm** $[mm]$			**Rotation - GE** [°]		
Weights	*Median*	*Min*	*Max*	*Median*	*Min*	*Max*
0.1	0.276	0.002	50.079	1.002	0.0	8.44
0.01	0.801	0.002	50.991	0.826	0.03	8.90
0.001	0.916	0.012	51.975	0.799	0.0	9.98

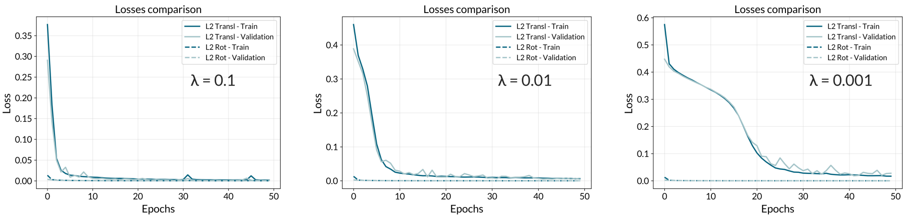

Fig. 2 Comparison of translation and rotation losses for the three tested values of the hyperparameter λ (0.1, 0.01, 0.001)

choose the best model weights considering the mean squared error obtained on the validation set (20% of the training set).

Figure 2 reports a comparison of translation and rotation losses for the three tested values of the hyperparameter λ.

Experiment 1

In this experiment, we investigated four different scenarios:

1. Training (p_1, p_2, p_3, p_4, 75088 images) and testing (p_5, p_6, 37544 images) on phantom data; initialisation with weights from ImageNet;
2. Inference on real data (real data with a gestational age of 23 weeks, r_1) using the model obtained in 1.1.
3. Training and testing on real data; initialisation with weights from 1.1. The model is trained on one fetus (r_1, 22029 images) with a gestational age of 23 weeks and tested on one of a second fetus with the same gestational age (r_2, 22029 images).

In cases 1.1 and 1.2, the test set was divided into two subgroups: a) planes acquired at random coordinates (Test RP) and b) planes acquired around the annotated transventricular standard plane (Test SP). Figure 3 reports the translation and rotation error distributions. Results for cases 1.1 and 1.3 are already reported in the paper. The median, maximum and minimum errors obtained for the regression of translation (Euclidean distance, in mm) and rotation (geodesic distance, in *degrees*) in Experiment 1 are shown in Table 3.
Additionally, we provide videos showing the outcome of the prediction of the plane pose within the volume in the build Unity environment for Experiment

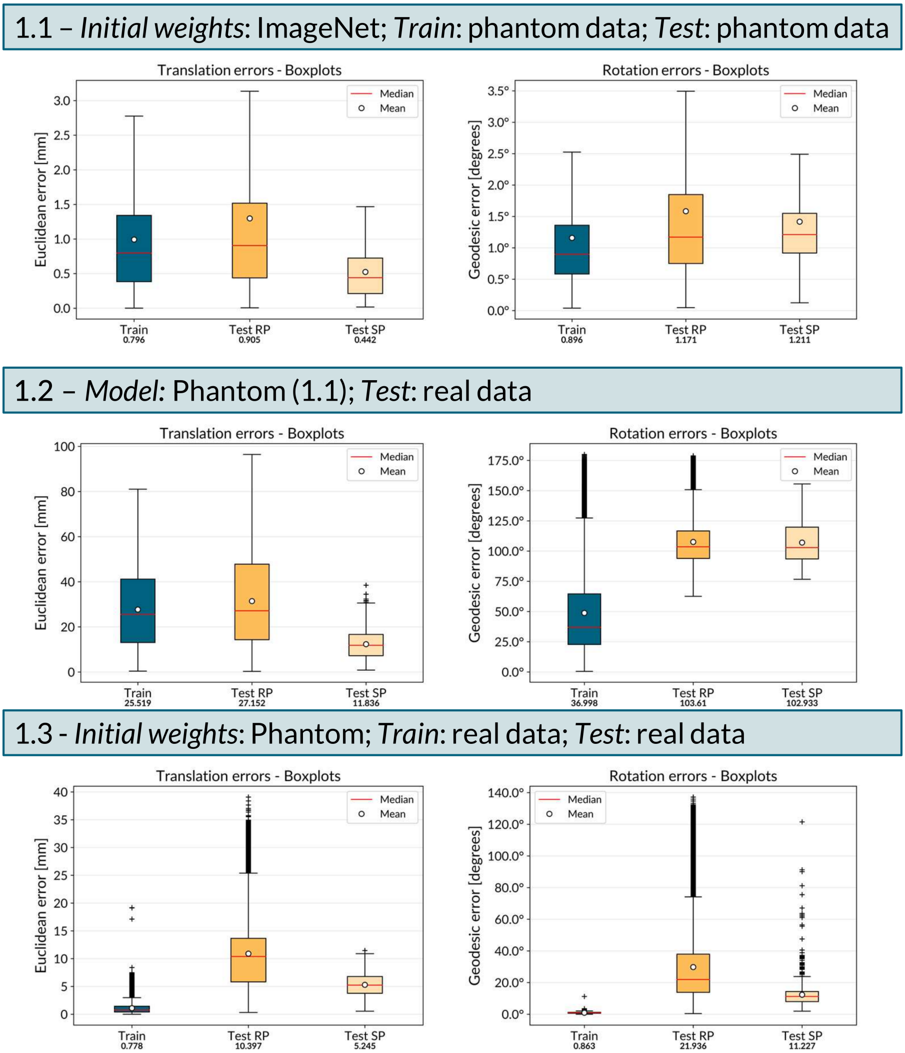

Fig. 3 Experiment 1 - Left: Translation and rotation error distributions in phantom (1.1) and real (1.2, 1.3) US data. In case 1.3. the training volume has a gestational age of 23 weeks, whereas the testing volume of 24 weeks.

1.1. In these videos, the green and orange edges surrounding the planes indicate the ground truth and the prediction, respectively. The names of the files equal the number of the experiment, namely 1.1_RP, 1.1_SP, where RP and SP refer to planes acquired at random coordinates and planes acquired around the annotated transventricular standard plane, respectively. The files can be found in the folder `videos`.

Table 3 Summary of the results obtained for translation and rotation. Norm: Euclidean distance, GE: Geodesic Error. p_j and r_i refers to the considered volume for phantom and real data, where i and j indicate the fetus and the acquisition number, respectively

				**Translation Norm [mm]**			**Rotation GE [deg]**		
Initial weights	*Training data*	*Testing data*	*Interval*	*Median*	*Min*	*Max*	*Median*	*Min*	*Max*
ImageNet	Phantom ($p_1, ...p_4$)	Phantom (p_5, p_6)	Test RP	0.90	0.01	53.47	1.17	0.04	20.85
			Test SP	0.44	0.02	10.43	1.21	0.13	137.78
Phantom	-	Real (r_2)	Test RP	27.15	0.29	96.45	103.6	62.62	178.8
			Test SP	11.84	0.86	38.46	102.9	76.71	155.58
Phantom	Real (r_1)	Real (r_2)	Test RP	10.39	0.32	39.08	21.94	0.43	137.21
			Test SP	5.24	0.55	11.46	11.23	1.92	121.5

Experiment 2

In addition, to verify that our model is able to generalise over different shapes and sizes of the fetus, as reported in the main paper, the trained model is tested on real data acquired on different fetuses ranging from a gestational age of 21 to 39 weeks. Here we report the dimension of the different volumes used for the test performed on different sizes and shapes of the fetal brain before the registration procedure ($coronal \times axial \times sagittal$, actual size of the acquired volumes as measured in 3D Slicer):

- Week 21: $207\times159\times134$ mm
- Week 22: $236\times203\times157$ mm
- Week 23: $211\times232\times153$ mm
- Week 24: $269\times180\times169$ mm
- Week 25: $271\times177\times168$ mm
- Week 39: $342\times307\times253$ mm

Additional Data

As introduced in the first section of the paper, we provide:

- The trained network (`model.py`) with the final weights for phantom (`phantom_weights.pt`) and real data (`real_weights.pt`). Model and weights can be found in the folder `model` and loaded as follows:

```
from model import *
saved_model = 'phantom_weights.pt'          # or 'real_weights.pt'

model = ResNet().to(device)
model.load_state_dict(torch.load(saved_model))
```

- The six 3D ultrasound phantom volumes. They can be found in the folder `volumes`. Their name are the same reported in the experiments: $\texttt{p}_1$`.vol`, $\texttt{p}_2$`.vol`, $\texttt{p}_3$`.vol`, $\texttt{p}_4$`.vol`, $\texttt{p}_5$`.vol`, $\texttt{p}_6$`.vol`. Note that to open this type of files in 3D Slicer the *SliceHeart* extension[1] is required. The 3D ultrasound

[1] SliceHeart extension (3D Slicer)

real volumes are external and can be obtained by following the instructions on their website[2] and signing a data transfer agreement.

- 2D slice of the annotated transventricular standard plane and the respective annotation for phantom data (`sp_`j`.png, poses.csv`, with $j = 1, ..., 6$). The files can be found in the folder `standard-planes`. Note that the coordinates were post-processed as follows:
  - Position values: normalisation between -1 and 1 using the `sklearn.preprocessing.MinMaxScaler` estimator[3];
  - Rotation values: conversion from *degrees* to *radians*.
- 2D sampled slices from the phantom 3D ultrasound volumes, and the respective pose annotations will be released after publication on the institutional website due to the large size.

[2] www.dataverse.nl
[3] MinMaxScaler, scikit-learn
